# Targeting mitochondrial ribosomal proteins: a functional and translational framework for neurodegenerative disease research

**DOI:** 10.3389/fneur.2026.1882474

**Published:** 2026-07-22

**Authors:** Luigi Del Giudice, Marco Guida, Maria Rosaria Aletta, Paola Pontieri

**Affiliations:** 1 Istituto Di Bioscienze e Biorisorse, Consiglio Nazionale Delle Ricerche, Sezione Di Napoli, Naples, Italy; 2 Dipartimento Di Biologia, Universita degli Studi Di Napoli Federico II, Naples, Italy; 3 Istituto Di Studi Sul Mediterraneo, Consiglio Nazionale Delle Ricerche, Naples, Italy

**Keywords:** Alzheimer's and Parkinson's disease, GEP3, mitochondrial disease, mitochondrial genome, MRPL44, NAM9, nuclear genome, yeast and C. elegans model organisms

## Abstract

Mitochondrial dysfunction is a central feature of neurodegenerative diseases, yet the molecular mechanisms governing mitochondrial protein synthesis remain insufficiently understood. Mitochondrial ribosomal proteins (MRPs), essential for the translation of mitochondrial-encoded components of the oxidative phosphorylation system, are emerging as critical regulators of neuronal homeostasis and survival. In this mini-review, we examine current knowledge on mitochondrial ribosomes with a focused analysis of three mitochondrial ribosomal proteins–MRPL44, NAM9, and GEP3–highlighting their structural and functional roles in maintaining mitochondrial integrity. We discuss evidence linking alterations in these proteins to key pathogenic processes relevant to neurodegeneration, including impaired oxidative phosphorylation, increased oxidative stress, and defective mitochondrial quality control. Importantly, we propose an integrative research perspective that positions these MRPs as potential modulators of tissue-specific vulnerability in neurodegenerative disorders. By synthesizing available data and identifying critical knowledge gaps, we outline future directions aimed at elucidating their contribution to neuronal dysfunction and disease progression. This work underscores mitochondrial ribosomal proteins as underexplored determinants of neurodegenerative pathology and suggests that their systematic investigation may reveal novel mechanistic insights and therapeutic opportunities.

## 1 Introduction

Mitochondrial diseases comprise a heterogeneous group of genetic disorders characterized by impaired mitochondrial function. Mitochondria are ubiquitous intracellular organelles, absent only in mature erythrocytes, and act as central hubs of intermediary metabolism. They are critically involved in multiple metabolic pathways, including oxidative phosphorylation (OXPHOS), fatty acid β-oxidation, the tricarboxylic acid (TCA] cycle, the urea cycle, gluconeogenesis, and ketogenesis (1).

Mitochondria generate more than 90% of the cellular energy required to sustain life and maintain organ function through ATP production. Impairment of mitochondrial function results in reduced ATP synthesis, leading to cellular dysfunction, injury, and potentially cell death. When this process occurs systemically, progressive multi-organ dysfunction may ensue (2). High-energy–demand tissues, such as the heart, brain, skeletal muscle, and lungs, are particularly vulnerable to mitochondrial dysfunction.

Mitochondrial diseases are clinically heterogeneous and often challenging to diagnose due to their highly variable presentation. Clinical manifestations may include seizures, stroke-like episodes, severe developmental delay, impaired ambulation, visual and speech deficits, gastrointestinal dysmotility, and a wide range of additional systemic complications. The involvement of three or more organ systems should raise suspicion of an underlying mitochondrial disorder (2). Although mitochondrial diseases have traditionally been considered pediatric conditions, adult-onset forms are increasingly recognized (3).

Mitochondrial biogenesis depends on the coordinated expression of two physically and functionally distinct genomes: nuclear DNA (nDNA) and mitochondrial DNA (mtDNA) (4, 5). Although an estimated ~3,000 nuclear genes contribute to mitochondrial structure and function, human mtDNA encodes only 37 genes. These include 13 polypeptides of the oxidative phosphorylation (OXPHOS) system and 24 RNAs (2 rRNAs and 22 tRNAs) required for intramitochondrial translation. All remaining mitochondrial proteins are nuclear-encoded, synthesized in the cytosol, and imported into the organelle. Beyond ATP production, mitochondria are central hubs of cellular metabolism, contributing to biosynthetic and catabolic pathways, redox balance, and nucleotide synthesis. Their functional specialization varies across developmental stages and differentiated tissues, underscoring their essential role in cellular homeostasis (6, 7).

Mitochondrial diseases result from inherited or *de novo* mutations in mitochondrial DNA (mtDNA) or nuclear DNA (nDNA), leading to defective mitochondrial proteins or RNA molecules. Despite the ubiquitous role of mitochondria, clinical manifestations are often tissue-specific, likely reflecting developmental and regulatory factors that remain poorly understood (7). Even considering tissue-specific isoforms, the marked variability in organ involvement across syndromes is difficult to explain. Because mitochondria perform diverse functions in different tissues, mitochondrial disorders comprise a highly heterogeneous group of conditions. Their clinical presentation is often variable and may complicate early diagnosis. A defining feature of these diseases is phenotypic heterogeneity: identical mtDNA mutations may produce different clinical phenotypes, whereas distinct mtDNA or nDNA mutations can result in similar syndromes, as exemplified by Leigh syndrome, which is associated with mutations in approximately a dozen different genes (8).

Mitochondrial diseases comprise a heterogeneous group of disorders caused by genetic defects affecting the assembly and/or function of oxidative phosphorylation (OXPHOS) complexes, impaired mitochondrial substrate or ion transport, or altered mitochondrial biogenesis. These defects reduce ATP production and result in highly variable clinical phenotypes, often requiring molecular diagnostics available only in specialized centers (1, 9). Ribosomal defects represent a relevant subset of these conditions, involving either cytoplasmic (80S) or mitochondrial (70S) ribosomes. They may be inherited or acquired and commonly arise from ribosomal protein haploinsufficiency or impaired ribosome biogenesis (10–12). Mutations in mitochondrial ribosomal proteins or assembly factors are particularly significant, as the mitochondrial ribosome translates the 13 essential OXPHOS subunits encoded by mitochondrial DNA. Consistently, several mitochondrial ribosomal protein genes are associated with disorders characterized by defective oxidative phosphorylation, including Leigh syndrome, multiple mitochondrial dysfunctions syndromes, and non-syndromic hearing loss (12).

In a recent study using the yeast *Saccharomyces cerevisiae* (S. cerevisiae) as a model organism to investigate human neurodegenerative diseases, three genes encoding mitochondrial ribosomal proteins—MRPL44, NAM9, and GEP3—were identified. Loss or dysfunction of these genes was associated with tellurite resistance and, as discussed in that study, may contribute to the pathogenesis of human neurodegenerative disorders such as Parkinson's and Alzheimer's diseases (13).

To date, direct evidence supporting an association between the nuclear-encoded mitochondrial proteins MRPL44, NAM9, and GEP3 and either Alzheimer's disease (AD) or Parkinson's disease (PD) in humans remains lacking. MRPL44 is a conserved mitochondrial large ribosomal subunit protein with a clear human ortholog. NAM9 is functionally related to mammalian mitochondrial small ribosomal subunit proteins, although a strict one-to-one orthology is not fully established. GEP3 is required for mitochondrial ribosome biogenesis in yeast, but no unambiguous human ortholog has been identified, despite the presence of functionally related factors in higher eukaryotes (14, 15).

However, given their emerging roles in mitochondrial biology and the central contribution of mitochondrial dysfunction to neurodegeneration, these proteins warrant further investigation as potential contributors to AD and PD pathogenesis. A key objective of this mini-review is therefore to highlight this unmet area of research and to stimulate future studies aimed at elucidating the relevance of MRPL44, NAM9, and GEP3 in human neurodegenerative disease.

Here, we propose an experimental strategy aimed at comprehensively characterizing the physiological functions of these three proteins, with particular emphasis on their roles in mitochondrial translation as well as their potential involvement in additional cellular processes beyond protein synthesis. This approach will provide a molecular framework for understanding the phenotypes associated with mutations in MRPL44, NAM9, and GEP3, which have already begun to be characterized in yeast, and will facilitate the extension of these investigations to other model systems. Ultimately, our goal is to elucidate how genetic defects affecting these proteins may contribute to the onset and progression of neurodegenerative disorders in humans and animals, and, on the other hand, to open a path to the production of drugs with therapeutic potential for the treatment of these diseases.

## 2 Current state of knowledge

**Figure 1:**
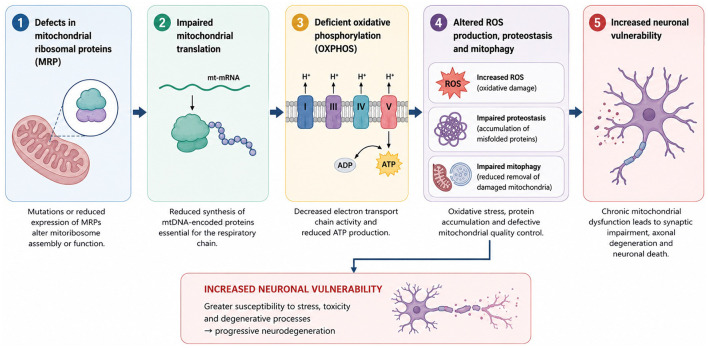
Schematic representation of the pathogenic cascade underlying mitochondrial ribosomal protein (MRP) defects associated with increased neuronal vulnerability, (*Original figure created by the authors using ChatGPT, OpenAI*).

The proposed pathogenic cascade linking mitochondrial ribosomal protein defects to neuronal vulnerability is summarized in Figure 1. Neurodegenerative diseases are characterized by the progressive and selective loss of anatomically or functionally related neuronal systems (14). Mitochondria, the main source of cellular ATP, are essential for neuronal function. Their dysfunction causes primary mitochondrial diseases and contributes to common neurodegenerative disorders, including Alzheimer's disease (AD), Parkinson's disease (PD), amyotrophic lateral sclerosis (ALS), and Huntington's disease (HD) (16–18). Although clinically and genetically heterogeneous, these disorders share mitochondrial dysfunction as a key pathogenic mechanism (16–18). Mitochondria regulate cell survival and death, influence aging, and interact with proteins implicated in inherited neurodegenerative diseases (15). Mitochondrial diseases arise from mutations in mitochondrial DNA (mtDNA) and nuclear DNA (nDNA), resulting in maternal, autosomal, or X-linked inheritance patterns. Traditionally defined by respiratory chain defects, the concept has expanded to include abnormalities in mitochondrial translation, lipid composition, dynamics (fission and fusion), and nuclear genes regulating mitochondrial homeostasis (17–20). Considerable progress has been made in understanding the molecular basis and genetic etiology of mitochondrial diseases, particularly through next-generation and whole-exome sequencing (NGS/WES) (21–24). These approaches provide insights into genes implicated in neurodegenerative disorders and clarify the role of mitochondrial dysfunction in their pathogenesis (24). Powerful genetic diagnostics now enable the analysis of thousands of patient genomes, facilitating molecular diagnoses in previously unresolved cases and uncovering novel genetic and pathogenic mechanisms (24). NGS has already transformed the diagnosis of nDNA-related mitochondrial diseases, increasing diagnostic yield from < 20% to >60% in some conditions (1).

Mitochondrial diseases result from inherited or *de novo* mutations in mtDNA or nDNA, affecting proteins or RNAs localized to mitochondria. Human cells harbor two genomes and translation systems: the nuclear genome (~3 × 10^9^ bp, 30,000–40,000 genes) translated by cytoplasmic ribosomes, and the mitochondrial genome (16,850 bp, encoding 13 mRNAs, 2 rRNAs, and 22 tRNAs). At least 1,500 nuclear-encoded proteins are imported into mitochondria (≈3%) (24–28). Cytoplasmic ribosomes comprise four rRNAs (28S, 18S, 5.8S, 5S) and 85 proteins; their genes are fully mapped and well characterized (24).

Mutations in genes encoding components of the cytoplasmic translation machinery—including ribosomal proteins and associated factors—have been linked to a variety of human diseases. Although mitochondrial translation defects can affect all tissues, accumulating evidence indicates that neurons represent a particularly vulnerable cellular population. This vulnerability likely reflects the convergence of high ATP requirements, dependence on oxidative phosphorylation, and limited regenerative capacity. Consequently, even modest reductions in mitochondrial protein synthesis may compromise respiratory chain biogenesis and trigger a cascade of bioenergetic, redox, and proteostatic disturbances that progressively impair neuronal function and survival. These observations support the view that defective mitochondrial translation is not merely a consequence of neurodegeneration but may constitute a primary pathogenic driver in several mitochondrial neurological disorders. Recent studies have further reinforced the concept that neuronal populations are exceptionally sensitive to perturbations of mitochondrial proteostasis and translation. Given their high energetic demands, reliance on oxidative phosphorylation, and long-lived post-mitotic nature, neurons are particularly vulnerable to defects in mitochondrial protein synthesis, which can compromise respiratory chain assembly, trigger integrated stress responses, and ultimately promote neurodegeneration (29–31).

**Figure 2:**
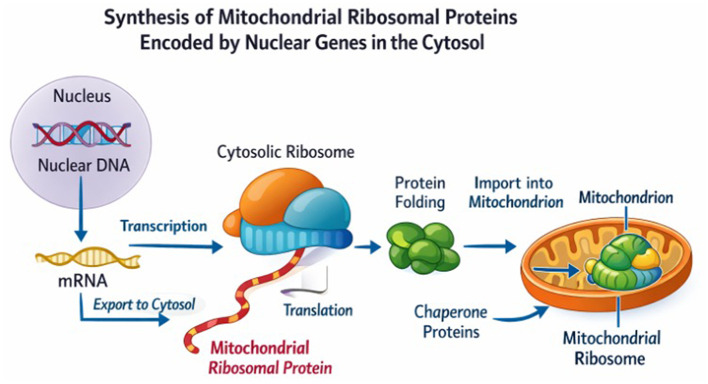
Schematic representation of mitochondrial ribosome biogenesis: mitochondrial rRNAs are encoded by mitochondrial DNA (mtDNA), whereas ribosomal proteins are encoded in the nuclear genome, translated on cytoplasmic ribosomes, and subsequently imported into mitochondria. (*Original figure created by the authors using ChatGPT, OpenAI)*.

In contrast, the components of the mitochondrial ribosome (mitoribosome) are encoded by both nuclear and mitochondrial genomes and are essential for the synthesis of the oxidative phosphorylation machinery. Mitoribosomes have been extensively studied in yeast, as a model system for eukaryotic cell biology, and in humans, due to their relevance to human health (25). Mitochondrial rRNAs are encoded by mitochondrial DNA (mtDNA), whereas the ribosomal proteins are encoded in the nuclear genome, synthesized on cytoplasmic ribosomes, and subsequently imported into mitochondria (Figure 2) (4). Inside the organelle, these proteins assemble with the two rRNAs to form functional mitochondrial ribosomes, which translate the 13 mtDNA-encoded mRNAs. Unlike cytoplasmic ribosomes and their bacterial ancestors, mitochondrial ribosomes are protein-rich rather than RNA-rich; for example, the human cytoplasmic ribosome contains approximately 7,500 bases of rRNA, compared with only ~2,000 bases in the mitochondrial ribosome, while both contain roughly 80 ribosomal proteins (26).

Regarding mitochondrial protein synthesis, mtDNA encodes 13 essential proteins required for oxidative phosphorylation, the process by which mitochondria generate ATP to meet the majority of the cell's energy demands for growth, differentiation, and development. Translation of the corresponding mRNAs occurs on the mitochondrial ribosomes. Interference with the synthesis of these proteins—whether through deletion or mutation of the mitochondrial genes encoding them, or of other components of the mitochondrial translation machinery such as mitochondrial tRNAs—is known to cause a range of mitochondrial diseases of variable severity, including myopathies and sensorineural disorders such as blindness and deafness (17). By analogy, the loss or mutation of any of the 78 proteins required for mitochondrial ribosome function (27) would also be expected to result in mitochondrial disease. Indeed, some genes encoding mitochondrial ribosomal proteins (MRPs) have been mapped to chromosomal loci already associated with human disorders, including deafness, retinitis pigmentosa, and Usher syndrome type 1E (28). Sequence information is now available for essentially all human MRPs, and advances in bioinformatics make it possible to implicate deficiencies in several additional MRPs as potential candidates for mitochondrial disease. With the advent of high-throughput sequencing, an increasing number of mutations in nuclear-encoded mitochondrial genes have been identified (32–39). For example, autosomal recessive mutations in translation elongation factors lead to phenotypes such as encephalopathy and cardiomyopathy (TSFM) or leukoencephalopathy (TUFM) (35). Mutations affecting mitochondrial translation have been identified in core mitochondrial ribosomal proteins (MRPS16, MRPS22, MRPL12) and accessory proteins (ObgH1, C7orf30) (35). MRPS16 and MRPS22 mutations destabilize the mitoribosome, while MRPL12 contributes to elongation factor recruitment and regulation of mitochondrial gene expression (35). Impaired translation can disrupt assembly of specific or all OXPHOS complexes, highlighting the tight regulation of mitochondrial translation (32–39). Over the past 5 years, advances in cryo-electron microscopy have revolutionized our understanding of mitoribosome structure and biogenesis, enabling the identification of novel factors involved in ribosomal subunit assembly and the regulation of mitochondrial translation. Concurrently, numerous studies have highlighted the pathogenic role of variants in genes encoding mitochondrial ribosomal proteins (MRPs) and assembly factors, linking them to a broad spectrum of multisystem disorders. In addition, growing interest has emerged in the contribution of mitochondrial dysfunction to neurodegenerative processes, particularly through alterations in bioenergetics, mitochondrial dynamics, mitophagy, and mitochondrial protein synthesis (40–44).

Mitochondrial dysfunction has been linked to the toxicity of heavy metals and metalloid oxyanions, including tellurium (Te), which is implicated in neurodegenerative disorders (45–52). Te exposure produces neurotoxic effects that vary with age: teratogenic hydrocephalus in fetuses and neonates, demyelinating peripheral neuropathy and central hypomyelination in weanlings, and cerebral lipofuscinosis with cognitive impairment in adult rats—a pattern reminiscent of Kuf's disease and Alzheimer's disease (47, 48). Te can directly impair mitochondrial energy metabolism, potentially contributing to neurodegeneration. Selenium deficiency, which may antagonize Te toxicity, in Alzheimer's disease brains further supports a role for Te in disease pathogenesis (47).

## 3 Proposed experimental strategy for the isolation and characterization of three mitochondrial ribosomal proteins

This research strategy investigates the expression and function of the mitochondrial ribosomal proteins MRPL44, NAM9, and GEP3 in human cells and in model systems, including yeast and *C. elegans*. using integrated cellular, molecular, and biochemical approaches (Tables 1–3).

**Table 1:** Objectives of the proposed experimental strategy.

Item	Description
a)	Generation of mutations in the MRPL44, NAM9 and GEP3 genes in human cells and in model systems using CRISPR/Cas9 technology;
b)	Isolation of mitochondria from wild-type and mutant human cells, as well as from wild-type and mutant model organisms;
c)	Microscopic, biochemical, and molecular characterization of the isolated mitochondria;
d)	Expression analysis of the MRPL44, NAM9 and GEP3 genes in a *Caenorhabditis elegans* Alzheimer's disease model strain expressing human tau protein;
e)	Production and purification of recombinant MRPL44, NAM9 and GEP3 proteins through cloning and heterologous expression in *Pichia pastoris* or in a *Saccharomyces cerevisiae nud1* mutant strain with enhanced protein export;
f)	*In vitro* synthesis of MRPL44, NAM9, and GEP3 proteins;
g)	Biochemical characterization of MRPL44, NAM9, and GEP3 proteins;
h)	Generation of specific antibodies against MRPL44, NAM9, and GEP3 proteins;
i)	Immunochemical analysis of MRPL44, NAM9, and GEP3 proteins produced *in vivo* in yeast or synthesized *in vitro*, using the corresponding specific antibodies;
l)	Cloning and transformation of tellurite-resistant Saccharomyces cerevisiae mutant strains with the MRPL44, NAM9, and GEP3 genes to restore wild-type tellurite sensitivity;
m)	Rescue of the healthy phenotype in human cells or in model systems (yeast, *C. elegans*) affected by mitochondrial neurodegenerative disorders.

**Table 2:** Description of the experimental activities outlined in Table 1.

Item	Description
a)	*Generation of mutations in MRPL44, NAM9, and GEP3 genes.* Mutations in the *MRPL44, NAM9*, and *GEP3* genes will be generated in human cells and in model organisms using CRISPR/Cas9 genome editing technology. Specifically, targeted modifications will be introduced into the coding sequences of each gene in human cell lines and in the selected model systems, including yeast, *C. elegans*, through CRISPR/Cas9-mediated gene editing.
b)	*Isolation of mitochondria from human and model organism cells.* Mitochondria will be isolated from human cells or from cells derived from the selected model organisms. Highly purified mitochondrial fractions will be prepared using either enzymatic protoplasting followed by differential centrifugation or a rapid mechanical disruption method based on glass bead homogenization.
c)	*Microscopic, biochemical, and molecular characterization of mitochondria in wild-type and mutant cells.* Mitochondria isolated from both wild-type and mutant human cells or model organisms will be characterized at the microscopic, biochemical, and molecular levels through: - Co-localization analysis by confocal microscopy. - Assessment of mitochondrial purity using marker enzymes, including succinate dehydrogenase, adenylate kinase, fumarase, and isocitrate dehydrogenase. - Functional evaluation of mitochondrial activity, including analyses of the Krebs cycle, the respiratory chain, and oxidative phosphorylation (OXPHOS). - Proteomic analysis for the systematic identification of mitochondrial proteins, lipidomic profiling to determine mitochondrial lipid composition, and metabolomic analysis to characterize mitochondrial metabolites.
d)	*Expression analysis of the modified MRPL44, NAM9, and GEP3 genes in a recombinant C. elegans Alzheimer's disease model expressing human tau protein.* The expression of CRISPR/Cas9-induced mutations in the MRPL44, NAM9, and GEP3 genes will be analyzed in a transgenic *Caenorhabditis elegans* strain expressing the human *tau* gene. Alternatively, *C. elegans* strains carrying CRISPR/Cas9-mediated modifications of MRPL44, NAM9, and GEP3 will be crossed with the transgenic *C. elegans* strain expressing human *tau*, which recapitulates the Alzheimer's disease phenotype. Gene expression levels will then be evaluated in the resulting recombinant progeny.
e)	*Production and purification of MRPL44, NAM9, and GEP3 proteins by heterologous expression in yeast systems.* The coding sequences of MRPL44, NAM9, and GEP3 will be cloned into appropriate expression vectors and transformed into *Pichia pastoris* or into a *Saccharomyces cerevisiae* nud1 mutant strain characterized by enhanced protein export. Recombinant proteins will be extracted and purified from transformed yeast cells using standard protein purification procedures.
f)	*In vitro synthesis of MRPL44, NAM9, and GEP3 proteins.* MRPL44, NAM9, and GEP3 proteins will be synthesized *in vitro* using cell-free expression systems starting from the corresponding coding sequences.
g)	*Biochemical characterization of MRPL44, NAM9, and GEP3 proteins.* MRPL44, NAM9, and GEP3 proteins will be characterized by mass spectrometric analyses (electrospray ionization mass spectrometry, MS/MS, and MALDI-TOF) to obtain information on peptide mapping, full-length amino acid sequences, and potential post-translational modifications of each protein. Partial results (N-terminal sequence) as well as final results (complete protein sequence) obtained from these biochemical procedures will be subjected to bioinformatic analysis against protein databases (Swiss-Prot, EMBL, etc.) using search algorithms (SRS, FASTA, BLAST) and alignment tools (ClustalX, etc.).
h)	*Production of antibodies against MRPL44, NAM9, and GEP3.* Polyclonal antibodies against MRPL44, NAM9, and GEP3 proteins will be generated in New Zealand White rabbits.
i)	*Immunochemical analysis of MRPL44, NAM9, and GEP3 proteins produced in vitro and in vivo in yeast.* MRPL44, NAM9, and GEP3 proteins produced both *in vitro* and *in vivo* in yeast will be analyzed by immunochemical assays using the corresponding specific antibodies. Quantitative and qualitative detection will be performed using a sandwich enzyme-linked immunosorbent assay (ELISA).
l)	*Cloning and transformation of tellurite-resistant S. cerevisiae mutant strains with the MRP*L4*4, NA*M9*, and GE*P3* genes to restore the wild-type tellurite-sensitive phenotype.* Tellurite-resistant *S. cerevisiae* mutant strains will be transformed with appropriate expression plasmids carrying the coding sequences of MRPL44, NAM9, and GEP3, respectively. The aim is to assess whether expression of each gene restores the wild-type tellurite-sensitive phenotype.
m)	*Recovery of the healthy phenotype in human cells and in model systems affected by mitochondrial neurodegenerative diseases.* The restoration of a healthy phenotype will be evaluated in human cells and in model organisms (yeast, *C. elegans*) affected by mitochondrial neurodegenerative diseases, such as Alzheimer's and Parkinson's disease. The recovery will be promoted through appropriate dietary and lifestyle interventions. In yeast and *C. elegans* models, dietary effects can be simulated by modifying the composition of growth media or feeding regimens. Furthermore, in yeast and *C. elegans*, feeding protocols can be designed to administer the mitochondrial ribosomal proteins MRPL44, NAM9, and GEP3, respectively, as candidate molecules aimed at targeting mitochondria and protecting neuronal cells from toxicity induced by mutant proteins.

**Table 3:** Expected outcomes of the proposed research strategy.

Item	Description
-	Generation and isolation of MRPL44, NAM9, and GEP3 mutant strains obtained through CRISPR/Cas9 genome editing in human cells or in model systems such as yeast, *C. elegans* exhibiting an Alzheimer-like phenotype.
-	Isolation and biochemical characterization of mitochondria from both wild-type and mutant strains of the above-mentioned human or model organism cells.
-	Crossing of MRPL44-, NAM9-, or GEP3-deficient *C. elegans* strains with a transgenic Alzheimer's disease *C. elegans* model expressing the human tau gene is expected to result in a rescue of locomotor defects associated with the Alzheimer-like phenotype.
-	Production of MRPL44, NAM9, and GEP3 proteins both *in vivo* and *in vitro*, followed by their biochemical and immunochemical characterization.
-	Restoration of wild-type sensitivity to tellurite in transformants of a tellurite-resistant *S. cerevisiae* mutant strain harboring the cloned MRPL44, NAM9, or GEP3 gene, respectively.
-	Development of a feeding protocol based on an appropriate diet and/or lifestyle intervention in human cells or model systems (yeast, *C. elegans*) aimed at restoring a healthy phenotype.
-	Establishment of a feeding protocol using purified mitochondrial ribosomal proteins (MRPL44, NAM9, and GEP3) as targeting and protective molecules to preserve mitochondrial function in yeast and *C. elegans* models exhibiting an Alzheimer-like phenotype and toxicity induced by mutant proteins.

## 4 Conclusions

A few years ago, Te was employed to investigate the prokaryotic origins of mitochondria (49). More recently, it has been used as an investigative tool in studies of neurodegenerative diseases, with *S. cerevisiae* serving as a model system (13). Through classical genetics, genomics, and molecular biology approaches, three nuclear genes were identified, encoding two mitochondrial ribosomal proteins (MRPL44 and NAM9) and one mitochondrial ribosome biogenesis factor (GEP3). Mutations in these genes have been associated with Parkinson's and Alzheimer's diseases (13).

Importantly, the Te compound enabled the identification of mitochondrial ribosomal proteins as a mechanistic link between gene dysfunction and neurodegeneration. In particular, mutations in these nuclear genes confer Te resistance in *S. cerevisiae* (49), while Te toxicity has previously been associated with the onset of neurodegenerative disorders (47, 48). These findings advance our understanding of mitochondrial ribosomal proteins involved in potassium tellurite resistance and provide a framework for investigating their potential contribution to neurodegenerative pathogenesis.

The identification of gene mutations in neurodegenerative diseases is reshaping our understanding of pathogenic mechanisms and enabling the development of targeted therapeutic strategies. Rigorous investigation of genetically homogeneous cohorts is essential to delineate natural history, as well as to establish robust biomarkers and longitudinal endpoints for clinical trials. Mitochondrial dysfunction is increasingly recognized as a convergent mechanism across clinically heterogeneous conditions, supported by expanding omics-based insights into nuclear–mitochondrial crosstalk.

Within this context, mitochondrial ribosomal proteins (MRPs) warrant focused investigation as potential primary determinants of disease or as genetic modifiers of established pathogenic variants (e.g., mt-tRNA and mt-rRNA mutations). *In vivo* models of mitochondrial translation defects will be critical to clarify these roles. Beyond their canonical function in translation, MRPs may also contribute to OXPHOS assembly, selective protein import, and the regulation of mitochondrial proteostasis (36, 39).

Overall, the evidence reviewed here highlights a field of growing scientific and clinical relevance. Despite significant advances, important knowledge gaps remain, particularly regarding the standardization of diagnostic criteria, prognostic stratification, and the identification of reliable biomarkers. In this context, the isolation and functional characterization of mitochondrial proteins—specifically MRPL44, NAM9, and GEP3—implicated in neurodegenerative mitochondrial diseases, appear both timely and highly relevant.

Continued mechanistic dissection of mitochondrial pathways is accelerating the development of mitochondria-targeted therapies (46–48), reinforcing the prospect that major disorders such as Alzheimer's disease, Parkinson's disease, cancer, and diabetes may become increasingly manageable in the near future (53).
